# Thioredoxin-Mimetic-Peptides Protect Cognitive Function after Mild Traumatic Brain Injury (mTBI)

**DOI:** 10.1371/journal.pone.0157064

**Published:** 2016-06-10

**Authors:** Renana Baratz-Goldstein, Hanna Deselms, Leore Raphael Heim, Lena Khomski, Barry J. Hoffer, Daphne Atlas, Chaim G. Pick

**Affiliations:** 1 Department of Anatomy and Anthropology, Sackler School of Medicine, Tel-Aviv University, Tel-Aviv, Israel; 2 Department Biological Chemistry, Institute of Life Sciences, The Hebrew University of Jerusalem, Jerusalem, 91904, Israel; 3 Department of Neurosurgery, Case Western Reserve University School of Medicine, Cleveland, Ohio, United States of America; 4 Sagol School of Neuroscience, Tel Aviv University, Tel Aviv, Israel; National Insttitute on Drug Abuse, UNITED STATES

## Abstract

Mild traumatic brain injury (mTBI) is recognized as a common injury among children, sportsmen, and elderly population. mTBI lacks visible objective structural brain damage but patients frequently suffer from long-lasting cognitive, behavioral and emotional difficulties associated with biochemical and cellular changes. Currently there is no effective treatment for patients with mTBI. The thioredoxin reductase/thioredoxin pathway (TrxR/Trx1) has both anti-inflammatory and anti-oxidative properties. If the system is compromised, Trx1 remains oxidized and triggers cell death via an ASK1-Trx1 signal transduction mechanism. We previously showed tri and tetra peptides which were derived from the canonical -CxxC- motif of the Trx1-active site, called thioredoxin mimetic (TXM) peptides, reversed inflammatory and oxidative stress damage mimicking Trx1 activity. Here, TXM-peptides were examined for protecting cognitive function following weight drop closed-head injury in a mouse model of mTBI. TXM-CB3 (AcCys-Pro-CysNH_2_), TXM-CB13 (DY-70; AcCys-Met-Lys-CysNH_2_) or AD4 (ACysNH_2_) were administered at 50 mg/kg, 60 min after injury and cognitive performance was monitored by the novel-object-recognition and Y-maze tests. Behavioral deficits subsequent to mTBI injury were reversed by a single dose of TXM-CB3, TXM-CB13 and, to a lesser extent, by AD4. TXM-CB13 similar to TXM-CB3 and AD4 reversed oxidative stress-induced phosphorylation of mitogen-activated kinases, p38^MAPK^ and c-Jun N-terminal kinase, (JNK) in human neuronal SH-SY5Y cells. We conclude that significantly improved cognitive behavior post mTBI by the TXM-peptides could result from anti-apoptotic, and/or anti-inflammatory activities. Future preclinical studies are required to establish the TXM-peptides as potential therapeutic drugs for brain injuries.

## Introduction

Traumatic brain (TBI) and spinal cord injuries are the largest causes of death and disability within the spectrum of trauma-related injuries. The Centers of Disease Control has termed TBI as a silent epidemic. TBI can be classified from mild to severe; most cases however are classified as mild traumatic brain injury (mTBI). TBI may lead to short and long term cognitive, emotional, and behavioral deficits and neuropsychiatric disorders [1]. mTBI is an increasingly common injury in modern society extending from military conflicts, to athletes, to everyday concussion injuries.

mTBI is a serious health care problem with limited available therapeutic intervention [2]. Similar to more severe traumatic brain injury [3], [4], [5], mTBI has been characterized as a multi- phase injury, comprised of a primary blunt force injury followed by secondary injuries lasting over an extended period of time [6], [7]. This delayed phase involves glutamate toxicity, permeability of the blood-brain-barrier (BBB), elevated oxidative stress, inflammation, astrocyte reactivity, apoptosis and other processes [8]. To develop an effective treatment for mTBI sequelae it is important to understand the delayed secondary molecular and biochemical cascades that occur after injury [9], [10]. Thus, secondary injury effects may potentially be reduced even if primary brain damage cannot be prevented [11].

A clinical study by Hoffer et al., demonstrated protection by Nacetyl-cysteine (NAC) treatment in U.S service members deployed to Iraq who had been exposed to a blast induced mTBI [12]. NAC as a thiol reagent and glutathione (GSH) precursor displays neuroprotective effects that are mediated by changes in reactive oxygen species (ROS), neuroinflammation, and glutamate flux across the membrane [13], [14]. These results support the view that oxidative stress, inflammation, mitochondrial stress, and glutamate transport play roles in neuronal cell death and behavioral outcomes from secondary blast effects [15], [16], [17], [18], [19]. Neuroprotection by NAC has also been shown in animal models of ischemia-reperfusion cerebral stroke [20], [13], [14], [21], and in a rodent closed head trauma model [22].

Since NAC does not penetrate cell membranes nor does it cross the BBB, we synthesized AD4 the amide form of NAC, also called NACA. AD4 was shown to increase GSH levels, reduce reactive oxygen species (ROS), lower inflammatory pathways by inhibiting MAPK phosphorylation, and inhibit MMP9 activity in vivo [23], [24]. AD4 also reduces ER stress and the unfolded protein response in Atm-/-thymocytes [25], and protects immortalized human brain endothelial-cells from methamphetamine-induced oxidative stress and toxicity [26]. Because the conversion of the carboxyl group of NAC to amide makes AD4 membrane permeable, this molecule was shown to be highly protective in different animal models of Parkinson’s disease [27], [28], and beta thalassemia [29]. All these results suggest that AD4 is a potential candidate for treating traumatic brain damages. Indeed, more recent studies have shown that AD4 improved mitochondrial bioenergetics through reducing oxidative stress in a controlled cortical impact model of TBI [30]. In these studies, impairment of cognitive function and behavioral outcome caused by oxidative damage at 7 days post-injury was improved by AD4 [30]. The neuroprotective effects of AD4 were demonstrated also following upper lumbar contusion spinal cord injury [31] and experimental focal penetrating brain injury in rats [32].

In the present study we tested, in the mTBI weight drop model in mice [33], [34], the effects of AD4 and di-thiol agents consisting of newly designed thioredoxin (Trx1)-mimetic peptide, called TXM-peptide [35]. Members of the TXM-peptides comprising of tri- and tetra-oligopeptides, are derived from the canonical -CxxC- motif of the Trx1-active site. They mimic the redox activity of Trx1, scavenge free radicals, prevent NF-kB nuclear translocation, and inhibit mitogen-activated protein kinases (MAPK). They exhibit higher potency and function at lower doses compared to NAC or AD4 [36], [35], [37], [38], [39]. TXM-CB3 (AcCysProCysNH_2_) and TXM-CB4 (AcCysGlyProCysNH_2_) were recently shown to be effective also as denitrosylation agents, reactivating S-nitrosothiol (SNO)-inhibited peroxiredoxin 1 [40]. TXM-CB3 and TXM-CB4 also rescue the human neuroblastoma SH-SY5Y cells and the rat insulinoma INS-1832/13 cells from S-nitrsoglutathione (GSNO)-induced growth inhibition [37] [40]. Here we show that TXM-CB3 and the novel TXM-CB13 (DY-70; AcCysMetLysNH_2_) improve cognitive function in a weight-drop closed-head injury mouse model of mTBI. A single dose of TXM-CB3 or DY-70 (50mg/kg) demonstrated an improved cognitive behavior 7 and 30 days post injury in two independent tests of cognition, the Y-maze and novel object recognition (NOR).

## Methods

### Reagents

Auranofin, triethylphosphine (2,3,4,6-tetra-O-acetyl-β-1-d-thiopyranosato-S) gold(I) (Enzo Life Sciences, Shoham, Israel); AD4 (NAC-amide) and thioredoxin mimetic peptides (TXM) TXM-CB3, were custom synthesized by Novetide, Ltd., Haifa, Israel; TXB-CB13(DY70) was from Biotech and Pharma Ltd, Israel. Tissue culture serum and medium were from Biological Industries, Kibbutz Beit-Haemek, Israel. All materials were purchased from Sigma, Jerusalem, if not otherwise stated.

### Animals

Male ICR mice (ages 6–8 weeks and weight 30–40 g) were initially purchased from HSD Jerusalem, Israel, and thereafter bred and maintained within the vivarium of Tel Aviv University, Israel. Mice were housed 4–6 per cage with ad libitum access to food (Purina rodent chow) and water on a 12:12 light/dark cycle at 22±1°C, with lighting during the light phase kept constant. Twice a week the cages were cleaned and the physical conditions of the mice were monitored. Animals weight was monitored once a week to ensure that there was no weight loss. All animals survived until the end of the experiment, with no signs of illness such as weight loss, changes in the fur, trembling or other signs of physical problems. All experimental manipulations were undertaken during the light phase of the cycle.

A minimum number of animals were used and all efforts were made to minimize potential suffering. Each animal was used for only one experiment. This study was carried out in strict accordance with the recommendations in the Guide for the Care and Use of Laboratory Animals of the National Institutes of Health. The protocol was approved by the Institutional Animal Care and Use Committees of Tel Aviv University Israel (M-15-031).

### Injury and drug administration

Mice were subjected to mTBI using a weight drop device as previously described [41]. Mice were anesthetized with isofluorene and then subjected to a traumatic brain injury using a weight drop of 50gm. All drugs were dissolved in saline and were administered 1 hour following the injury via IP injection. AD4 was used at 100mg/kg, and 50 mg/kg. TXM-peptides were evaluated at 50 mg/kg. The Y-maze test to evaluate spatial learning and the Novel object recognition test to evaluate visual learning were assessed 7 or 30 days post injury (See below). These doses are based on previous literature detailed in the Introduction (ref [38], [39]). Carrying out dose response studies would have required use of many more animals which somewhat conflicts with the ethical requirements to use the minimum number to establish the veracity experimental findings.

### Cognitive behavioral tests

Two behavioral paradigms were used to evaluate changes in cognition, the Y-maze and the novel object recognition (NOR). The details of these two tests are the same as those described in our previous papers [22], [41] below to facilitate reproducibility by other investigators.

#### Y-maze test

Spatial memory was assessed using the Y-maze [42]. The Y-maze was constructed of black Plexiglas with three identical arms (30 × 8 × 15 cm each at an angle of 120° from the others). This task takes advantage of a rodent’s preference to explore novel rather than familiar places. The test included two trials separated by a two-minute interval during which the mouse was returned to its home cage. The first trial, "familiarization", was five minutes long with only two arms open (the start arm and the designated "old" arm), and the third arm blocked by a door (the “novel” arm). The second trial lasted two minutes with all three arms open and the time spent in each of the arms was quantified. Discrimination of spatial novelty was assessed by a preference index [43] determined as: (time in the new—time in the old arm)/(time in the new + time in the old arm). Between each run and between each mouse the maze was cleaned with 70% ethanol.

#### The novel object recognition test (NOR)

An object recognition test was used to evaluate recognition memory [44]. This task is based on the tendency of rodents to explore a new object. The open field was constructed of black Plexiglas (59 x 59 cm arena surrounded by 20 cm high black walls). The task includes 3 trials of 5 min separated by 24-hour intervals. On the first day the mice were placed in an empty arena for habituation. On the second day the mice were placed into the arena with two identical objects, A and B, positioned 40 cm from each other and 10 cm from the walls. On the third day the mice were placed into the arena with object A (the same as on the second day) and object C (a new object). The arena and the objects were cleaned with 70% ethanol between each trial. Exploration of an object was defined as rearing on the object or sniffing it at a distance of less than 2 cm and/or touching it with the nose. Discrimination of recognition novelty was assessed by a preference index [43], (time exploring the new object—time exploring the old object)/(total time exploring an object). Mice that spent less than 10% of the total time were excluded from the analysis.

### Data analysis

All behavioral results are presented as mean ± SEM and were analyzed with SPSS 23 software (Genius Systems, Petah Tikva, Israel). One-way ANOVA’s were performed to compare between all groups, followed by LSD post hoc tests.

### Cells

Human neuroblastoma SH-SY5Y- cells were kindly provided by H Soreq (Hebrew University of Jerusalem, Israel). The cells were cultured in DMEM/F12 HAM 1:1 medium supplemented with 10% fetal bovine serum (FBS) and penicillin–streptomycin, incubated at 37°C with 5% CO_2._

### MAPK activity in human neuronal SH-SY5Y cells

The anti-inflammatory activity of TXM-CB13 (DY70) was tested essentially as previously reported for TXM-CB3 [39]. SH-SY5Y cells were treated for 30 min with a 5μM auranofin (AuF), an inhibitor of thioredoxin reductase, washed and incubated for 2h with or without TXM-CB13 (DY70) at the indicated concentrations. After washing with one ml of PBS the cells were lysed in 0.12 ml lysis buffer (150mM Tris, pH 6.8, 10% Glycerol, 0.6% SDS, Bromophenol Blue, supplemented with 7μl β-Mercaptoethanol/ml). The protein concentration of the lysates was determined by using Coomassie Brilliant-Blue staining. Cell lysates were heated to 100°C for 5 min prior to separation by SDS gel electrophoresis.

### Western blot analysis

The phosphorylation of MAPKs was monitored by Western blot analysis using selective antibodies against phosphorylated p38^MAPK^, JNK (c-Jun N-terminal kinase), and ERK1/2, and the corresponding non-phosphorylated MAPKs (see below). We used the housekeeping protein Glyceraldehyde-3-phosphate dehydrogenase, GAPDH, to normalize the extent of phosphorylation.

Protein samples (20–30μg) were loaded on 10% SDS-PAGE. After separation by electrophoresis the proteins were transferred electrophoretically to nitrocellulose membrane (Whatman, Germany). The blots were blocked by incubation for 1 h at RT in 25 mM Tris–HCl pH 7.4, 0.9% NaCl and 0.02% Tween-20 with 4% Difco skim milk (BD, USA). The blots were incubated overnight at 4°C with the corresponding primary antibody: p-SAPK/JNK (Thr183/Tyr185), rabbit mAb; SAPK/JNK; pERK1/2 (Thr 202/Tyr204), mouse mAb; ERK2 (Santa Cruz, U.S.A) rabbit Ab; mouse mAb; p-p38MAP kinase (Thr180/Tyr182), rabbit mAb; p38, rabbit Ab; Antibodies were obtained from Cell Signaling Tech. (USA), and used at 1:1000. Rabbit Ab; GAPDH (Glyceraldehyde 3-phosphate dehydrogenase) and ERK2, polyclonal rabbit Ab obtained from Santa Cruz, (USA). Proteins were detected with anti-mouse or anti-rabbit IgG-HRP linked antibody 1:10,000; Cell Signaling, Tech. (USA) and calibrated with the housekeeping protein Rabbit Ab; GAPDH. Immunoblots were developed by direct capture of chemiluminescence with the DNR MF-ChemiBIS 3.2 Bio-Imaging System. For data analysis, the amounts of each band were quantified by using the EZ-Quant software (version 2.2) and plotted using a linear regression program.

## Results

### Protection of spatial and visual learning by AD4

**Fig 1:**
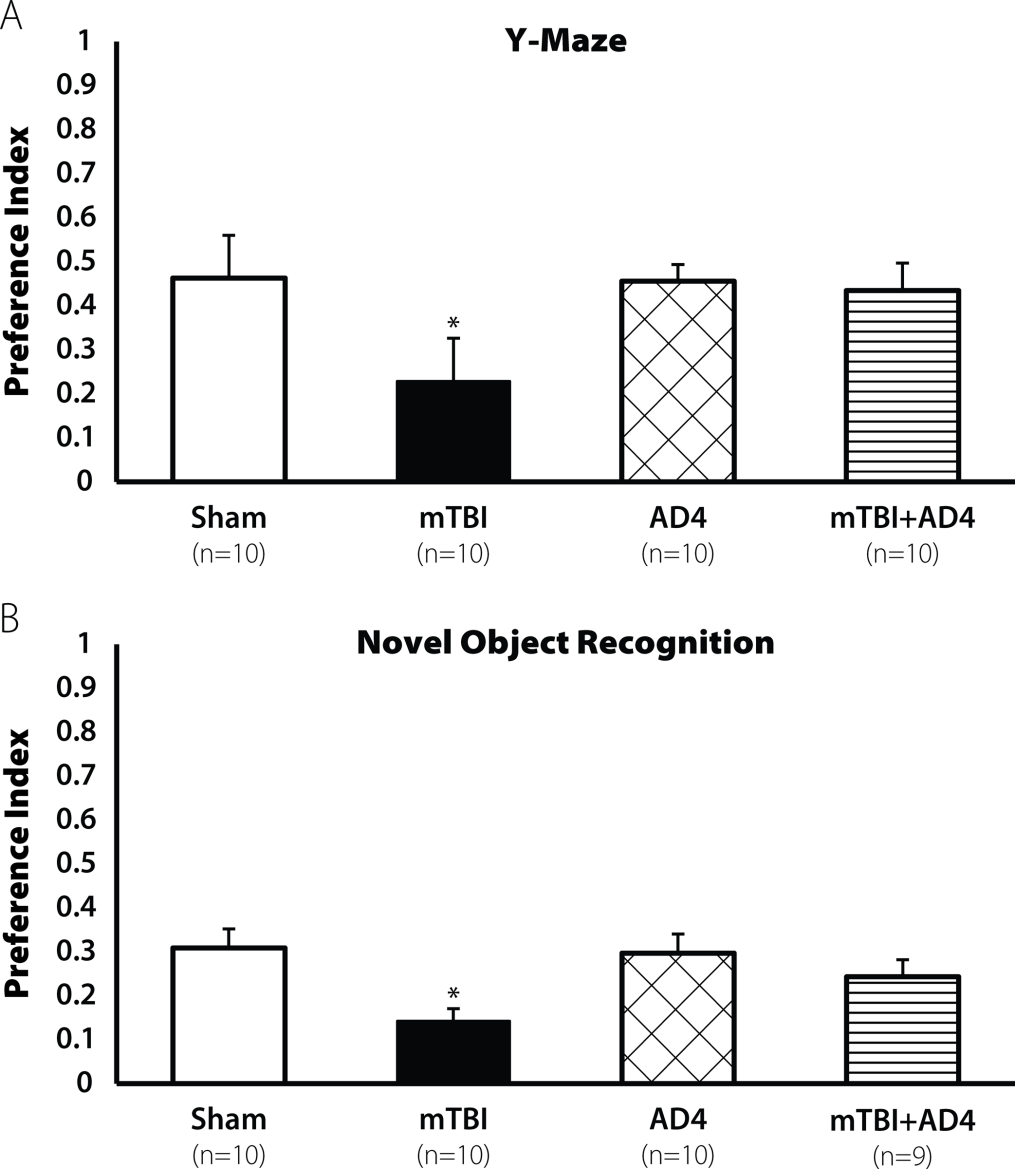
Restoration of learning ability by AD4 (100mg/kg) 7 days post injury. Seven days post injury mTBI mice exhibit lower learning ability both (**A**) in the Y-maze test and in **(B**) the NOR test (*p<0.05). Treatment with AD4 (100mg/kg) improved this impairment, revealed by LSD post hoc (p<0. 01). Values are mean ± SEM.

Mice were subjected to mTBI using a weight drop as previously described (See Methods). One hour later a group of mice were injected intraperitoneally (i.p) with AD4 at a dose of 100mg/kg, a dose based on previous literature. Seven days post injury we found a decline in spatial learning in the Y-maze test [F(3,39) = 3.862, p<0.05, **Fig 1A**] in vehicle treated animals. No decline was observed in the AD4-treated mice. LSD post hoc confirmed that the mTBI group was different from all other groups (p<0.05). We also found a decline in visual learning ability in the novel object recognition test [F(3,38) = 3.682. p<0.05, **Fig 1B**]. LSD post hoc analysis confirmed that the mTBI group was different from the AD4-treated groups (p <0.05).

**Fig 2:**
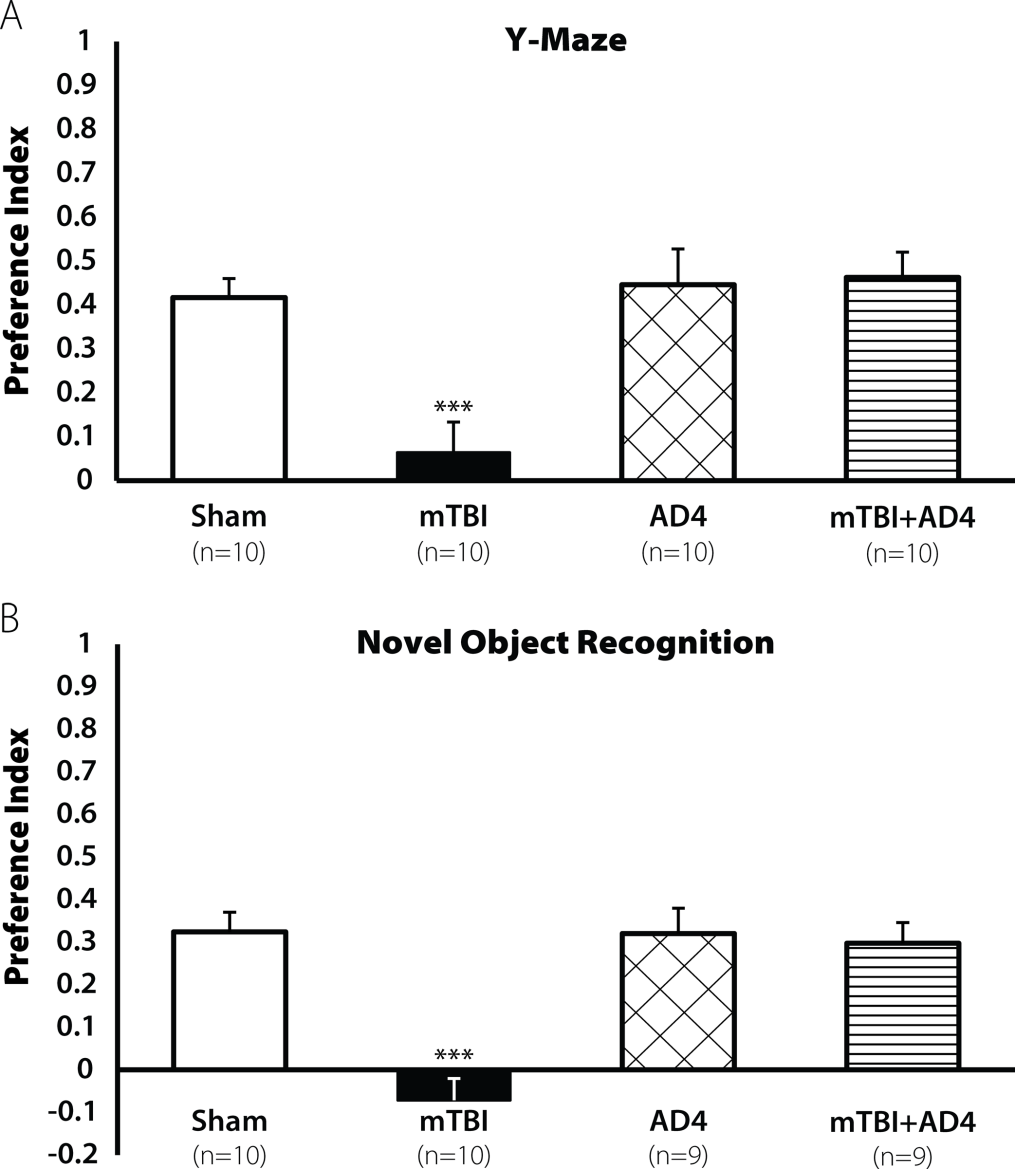
Restoration of learning ability by AD4 (100 mg/kg) 30 days post injury. Thirty days post injury, the mTBI mice showed a decline in cognitive performance both in **(A**) the Y-maze test and in the (**B**) NOR test (*p<0.001). Treatment with AD4 (100mg/kg) improved these impairments, revealed by LSD post hoc (p<0.001). Values are mean ± SEM.

The cognitive impairments were observed also 30 days post injury both in the Y-maze test [F(3,37) = 8.701, p<0.001; **Fig 2A**] and in the novel object recognition test [F(3,35) = 13.771, p<0.001; **Fig 2B**]. AD4-treated groups showed no decline in cognitive ability. LSD post hoc analysis confirmed that the mTBI group displayed lower cognitive ability compare with all other groups (***p <0.001, **p<0.001, respectively).

### Protection of spatial and visual learning by TXM-Peptides

**Fig 3:**
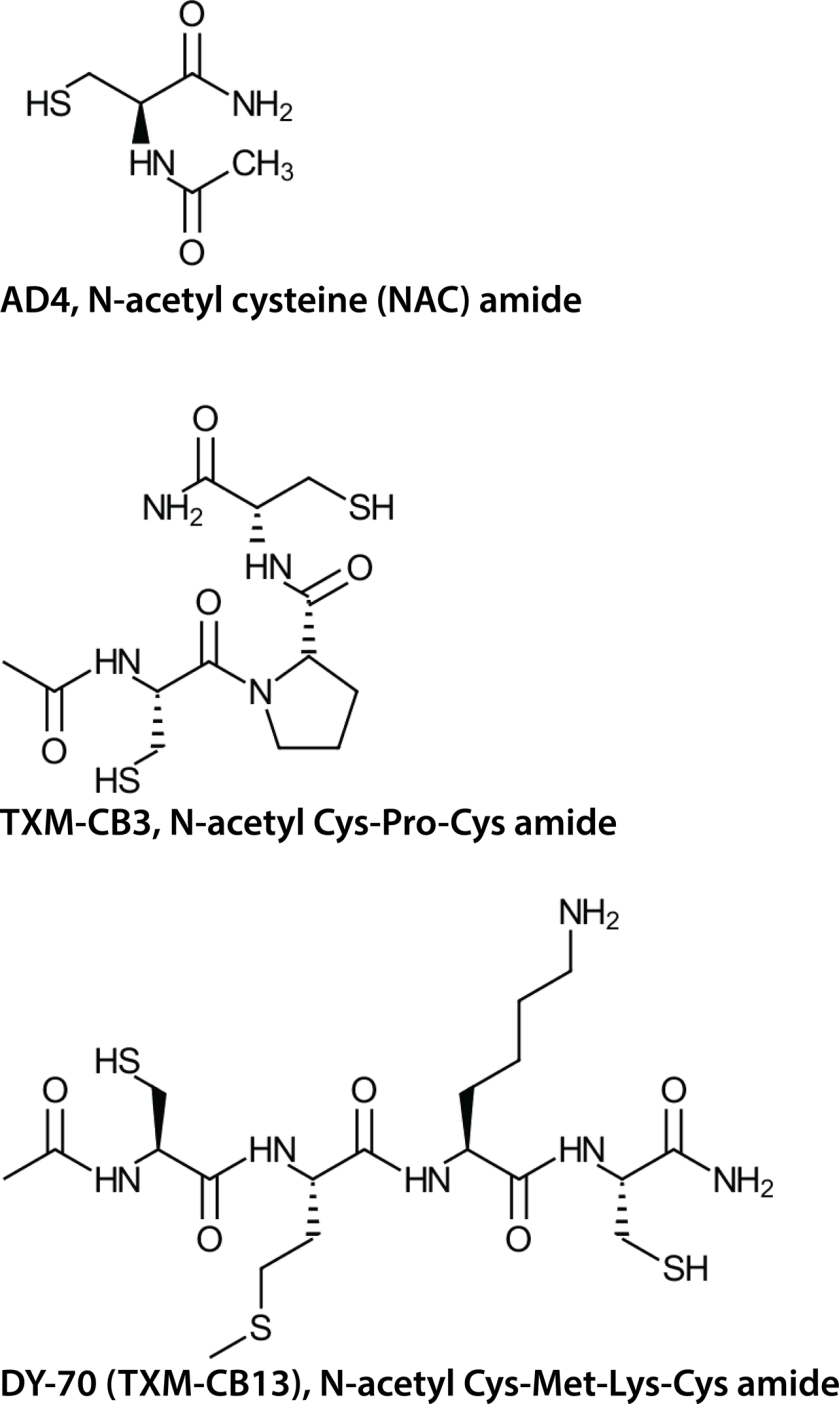
The chemical structures of AD4 (top), TXM-CB3 (middle), and DY-70 (bottom).

Unlike the single Cys residue of NAC or AD4, TXM-peptides harbor two Cys residues (**Fig 3**). They were shown to be more potent than NAC or AD4 in reversing auranofin or high glucose-induced oxidative stress [35], [37], [39].

As shown above, AD4 at 100 mg/kg showed a significant protection of cognitive ability following mTBI. Based on this potent mitigation of cognitive impairment, in further subsequent studies the dose of AD4 was lowered to 50 mg/kg to avoid ceiling effects for testing in comparison to 50 mg/kg of TXM-peptides.

TXM-CB13 (DY-70) a tetra-peptide comprising a–CxxC- motif, and TXM-CB3 comprising a -CxC- motif, were tested on cognitive behavior. Previous in vivo studies showed that TXM-CB3 lowered inflammation in the brain of diabetic rats [39] and decreased the number of inflammatory cells and airway hyper responsiveness in the lungs in a mouse model of asthma [38]. The novel TXM-CB13 (DY70) used in the present study contains of Met and Lys within the -C-x-x-C- motif. These amino acids, act as precursors of carnitine. Neuroprotection by brain acetyl carnitine has been demonstrated in various neurological diseases. Acetyl-carnitine improves mitochondrial function, increases antioxidant activity, and was reported to be beneficial in Alzheimer’s and Parkinson’s animal models if combined with alpha-lipoic acid [45], [46], (See review [47]).

**Fig 4:**
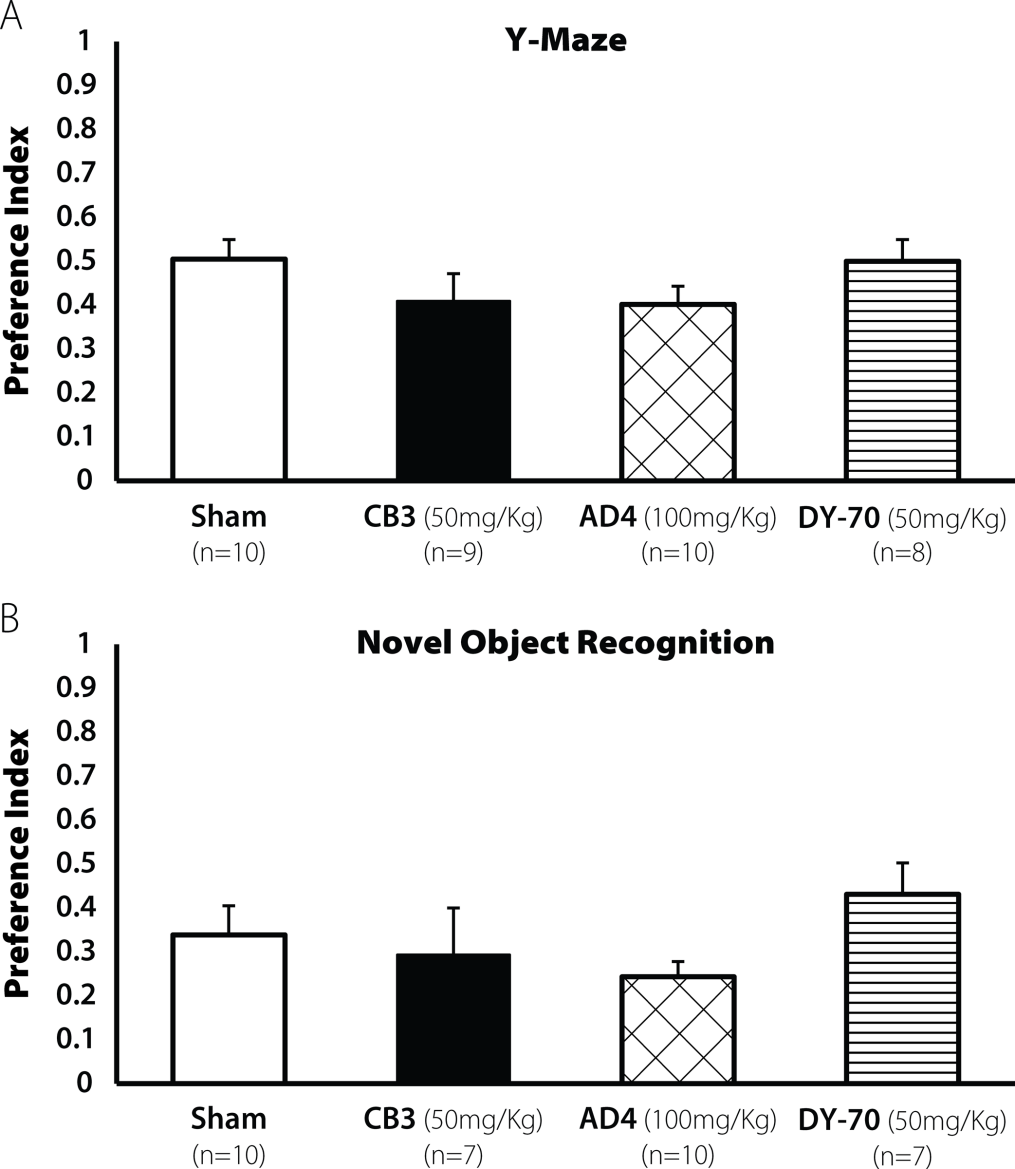
Administration of thiol reagnets alone did not affect leaning ability 30 days post injection. Mice administered AD4 and the TXM-peptides did not induce any learning deficit. All groups showed the same performance as the sham group **(A**) Spatial learning was tested using the Y-maze (N.S) and (**B**) Visual learning was tested using the NOR test (N.S). Values are mean ± SEM.

Administration of AD4, TXM-CB3 or TXM-CB13 alone (in the absence of TBI) revealed no changes in cognitive behavior when compared with the sham group in the Y-maze test [F(3,36) = 0.712, N.S; **Fig 4A**], and in the NOR test [F(3,33) = 0.587, N.S; **Fig 4B**] 30 days after administration. Because we did not see any effect 30 days post administration we did not explore the influence of the drugs alone at 7 days.

**Fig 5:**
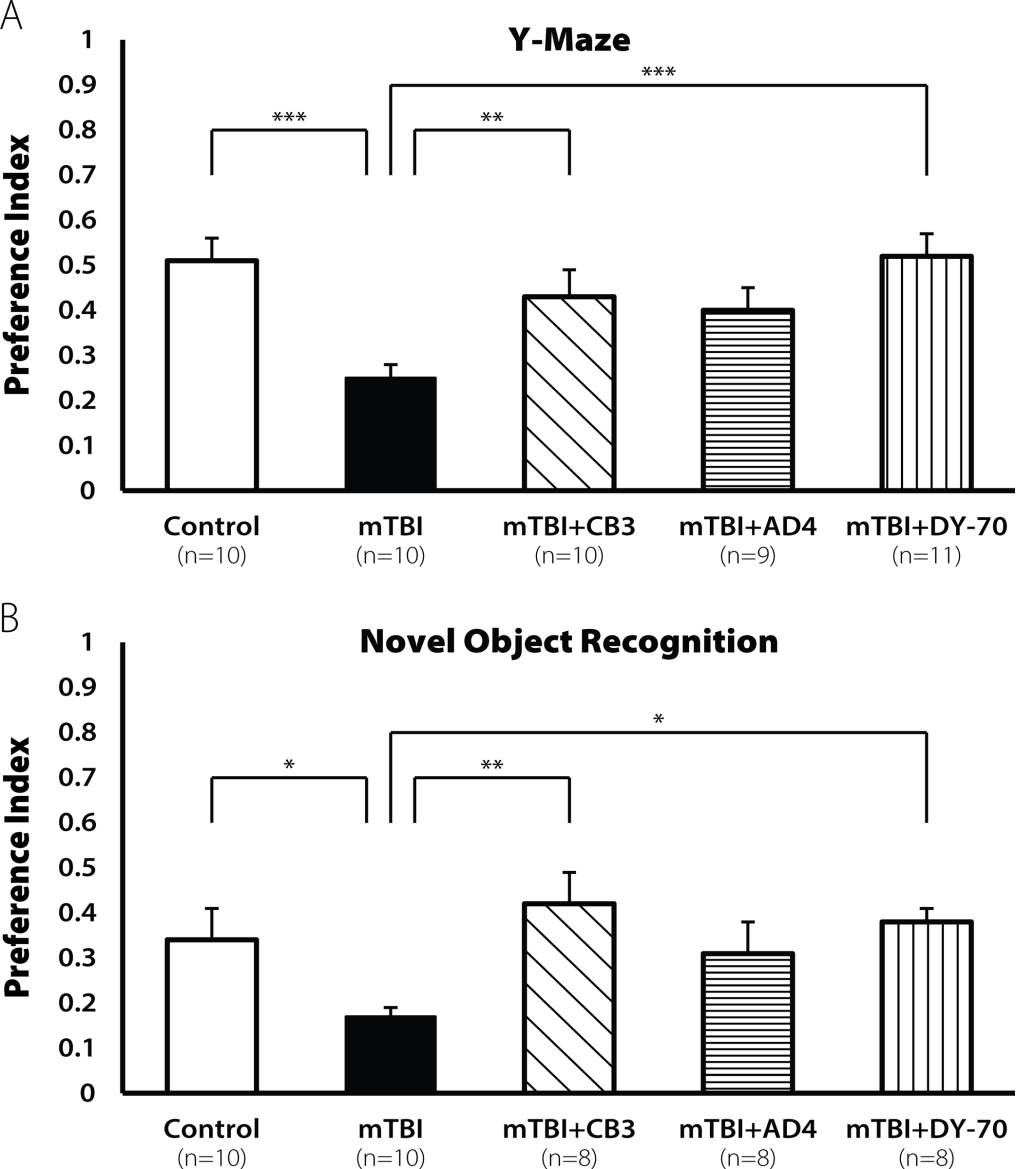
Restoration of learning ability by TXM-CB3, AD4, and DY-70 (50mg/kg) 7 days post injury. Seven days post injury, the mTBI mice showed a decline in cognitive performance both in the (**A**) Y-maze test and in the (**B**) NOR test (***p<0.001, *p<0.05). Treatment with TXM-CB3 and DY-70 (50mg/kg) improved and AD4 did not improve the cognitive performance, revealed by LSD post. Values are mean ± SEM.

AD4 and TXM-peptides were administered at a 50 mg/kg dose 60 min post injury. Seven days post injury the mTBI group showed a decline in cognitive performance in the Y-maze test [F(4,49) = 4.524, p < 0.01; **Fig 5A**]. LSD post hoc analyses confirmed that the mTBI group was significantly different from the sham group (p <0.001). Treatment with TXM-CB3 or DY-70 significantly improved this behavioral deficit (p <0.005, p <0.001, respectively). The lower dose AD4 treatment produced a trend to mitigate cognitive impairment (p = 0.07).

Decline in learning ability of the mTBI group was detected also in the novel object recognition test [F(4,46) = 4.524, p <0.05; **Fig 5B**]. LSD post hoc analyses confirmed that the mTBI group was significantly different from the sham group (p <0.05). Treatment with TXM-CB3 or DY-70 improved this behavioral deficit (p <0.005, p <0.05, respectively).

**Fig 6:**
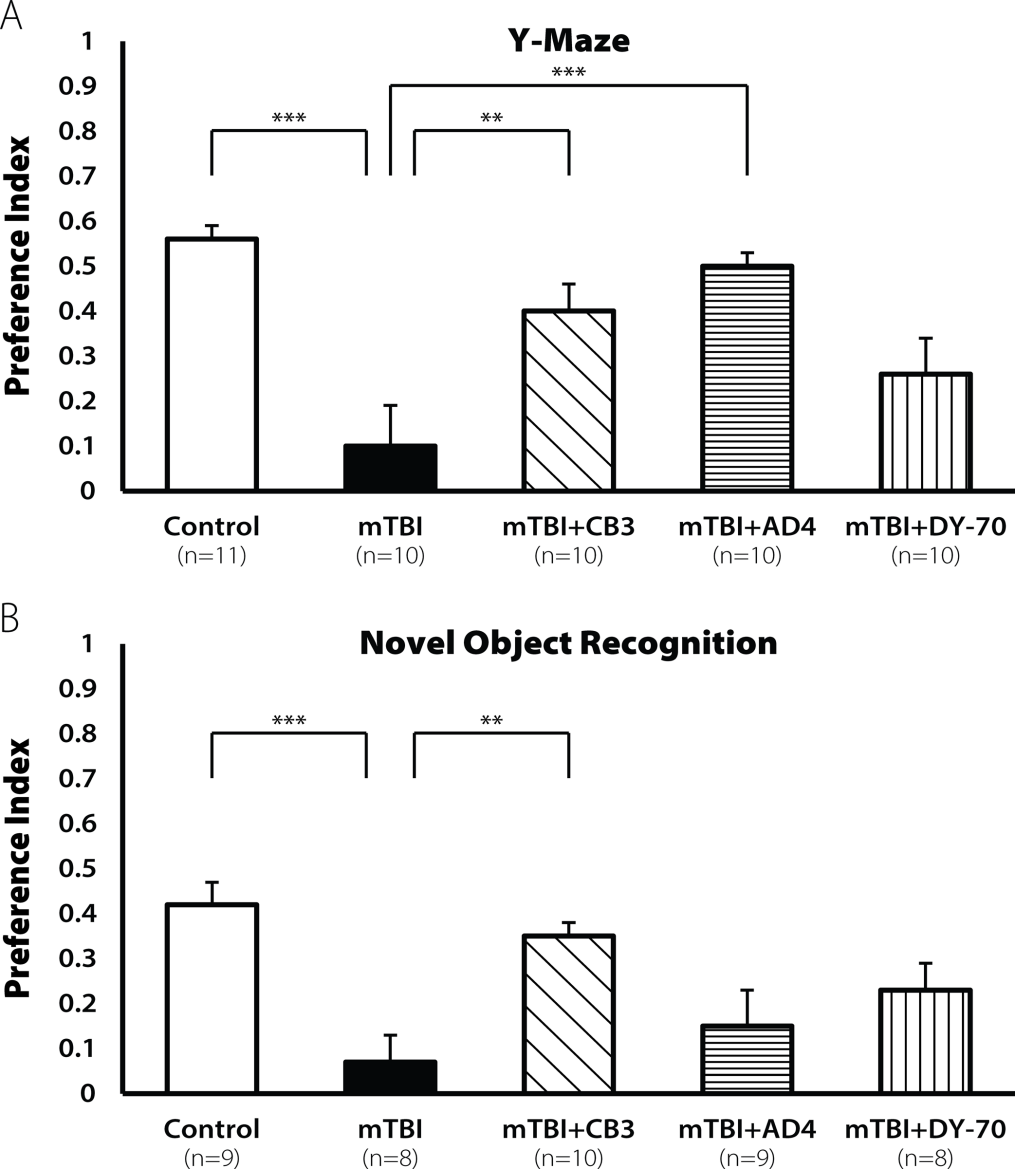
Restoration of learning ability by TXM-CB3, AD4, and DY-70 (50mg/kg) 30 days post injury. Thirty days post injury the mTBI mice showed a decline in cognitive performance both in (**A**) Y-maze test and in the (**B**) NOR test (***p<0.001). Treatment with TXM-CB3 (50 mg/kg) improved the cognitive performance. DY-70 (50mg/kg) did not improve the cognitive performance, revealed by LSD post. AD4 (50mg/kg) improved performance only in the Y maze test. Values are mean ± SEM.

Thirty days post injury the mTBI mice exhibited lower learning ability in the Y- maze test [F(4,51) = 7.007, p<0.001; **Fig 6A**]. LSD post hoc analyses confirmed that the mTBI group was significantly different from the sham group (p <0.0001). Treatment with TXM-CB3 or AD4 improved the behavioral deficit (p <0.005, p <0.001, respectively).

A cognitive decline was found also in the novel object recognition [F(4,45) = 4.682, p <0.001; **Fig 6B**]. LSD post hoc analyses confirmed that the mTBI group was significantly different from the sham group (p <0.001). Only treatment with TXM-CB3 improved this behavioral deficit (p <0.005), whereas AD4+DY-70 treatment showed no improvement.

### The anti-inflammatory/anti-apoptotic activity of DY-70 (TXM-CB13) in neuronal cells

Next, we induced oxidative stress in SH-SY5Y cells, a human neuroblastoma cell line and tested the effects of DY-70 on activation of the inflammatory pathways. This immortal cell line has neuronal features and hence is often used as a cellular model of various neurological oxidative stress related disorders. We monitored the phosphorylation/activation of p38^MAPK^, JNK, and ERK1/2 induced by exposing the cells to auranofin (AuF), a TrxR selective inhibitor (see Methods). Trx1 in the reduced state is associated with a complex with the apoptosis signal-regulating kinase1 (ASK1) and prevents activation of apoptotic pathway [48], [37]. In the oxidized state Trx1 dissociates from the complex and the freed ASK1 activates the MAPK-apoptotic cascade. Oxidative stress was generated by AuF that prevents reduction of oxidized Trx1 back to the active reduced state and leads to dissociation of ASK1.

**Fig 7:**
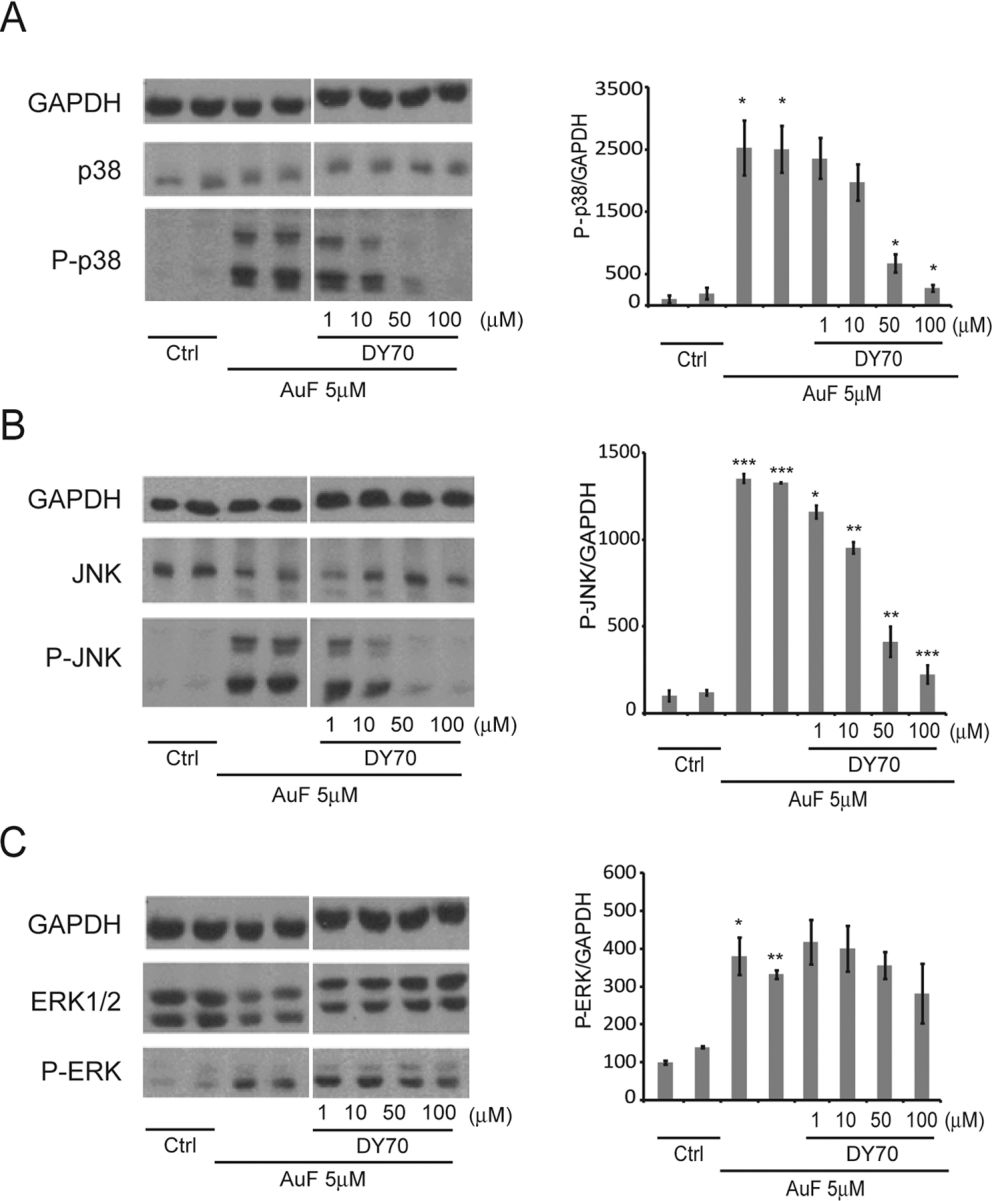
Trx1 mimetic peptide TXM-CB13 (DY-70) reverses the auranofin-induced phosphorylation of JNK and p38^MAPK^ in SH-SH5Y cells. SH-SY5Y cells were treated with 7.5μM AuF for 30 min, washed, and treated with or without increasing concentrations of DY-70. Lysates were analyzed by immunoblotting with the indicated antibodies after separating proteins on SDS-PAGE. MAPK phosphorylation of (**A**) p38 (**B**) JNK or (**C**) ERK1/2 were visualized by immunoblot (*left*). The ratios of pp38, pJNK and ppERK1/2 to the housekeeping GAPDH protein were calculated based on three independent experiments (*right)*. The values shown are averages (±SEM) of three independent experiments normalized to the phosphorylation state of cells treated with AuF after 4.5hr. Student's *t*-test (two populations) was performed for AuF treated cells. *P value <0.05; **P value <0.01; ***P value < 0.005.

SH-SY5Y cells were treated for 30 min with 5μM AuF washed and incubated for 2h with or without DY-70 at the indicated concentrations. The phosphorylation of MAPKinases was monitored by Western blot analysis using the corresponding anti phosphorylated p38^MAPK^, JNK, and ERK1/2 antibodies, and normalized to total GAPDH using selective anti GAPDH antibodies (**Fig 7A–7C**). The reduction of AuF-induced JNK and p38^MAPK^ phosphorylation was concentration-dependent (**Fig 7A and 7B**). DY-70 was considerably more effective in lowering AuF-induced JNK and p38^MAPK^ phosphorylation compared to AuF-induced ERK1/2 phosphorylation (**Fig 7C**). This result is consistent with a smaller effect of TXM-CB3 on ERK1/2 phosphorylation compared to JNK and p38^MAPK^ phosphorylation, previously reported in insulinoma cells [37] and in the brain of the Zucker Diabetic Fatty (ZDF) rat [39].

## Discussion

Although basic and clinical research in traumatic brain injury (TBI) have been focused on understanding the biological process of the disorder and the development of advanced diagnostic tools, few reported treatments have shown a decrease in secondary brain injury. Blast-related inflammatory effects have been shown to last weeks or months following exposure and are one of the hallmarks of TBI. More recently, neuroinflammation has been shown to be responsible for many of the secondary effect on cognitive behavior. Following brain injury an inflammatory response provides protection, but if it persists beyond the initial defensive/reparative and beneficial functions, it might lead to secondary injuries. These may in turn lead to the decline in cognitive processes. There is an unmet need for decreasing the onset and the ensuing low-grade inflammation caused by TBI. Potential treatments of TBI are aimed mainly at minimizing secondary brain injuries.

Among the processes that contribute to the aggravation of TBI outcome are inflammatory processes, mitochondrial damage, and oxidative stress-related damage including free radical accumulation [49], [50]. Following TBI there is a widely recognized inflammatory reaction, including elevation of inflammatory cytokines, and chemokines. It was suggested that inhibition of an inflammatory response could be important for promoting various protective mechanisms in response to this insult [51]. Long lasting inflammatory reactions might result in neuronal dysfunction and degeneration by inducing a self-propagating pathological cascade [52].

In the present study we showed the effects on cognitive behavior after mTBI of a single dose treatment of AD4 (NAC-amide; NACA), TXM-CB3, or TXM-CB13 containing the–CxC- and–CxxC-catalytic motifs of Trx1, respectively. All these thiol reagents administered 60 min post trauma, restored cognitive function following mTBI. As noted above, the concentrations of AD4 and TXM-peptides were chosen based on our previous in vivo studies. AD4 showed efficacy at 60–120mg/kg in attenuating airway inflammation and hyperresponsiveness in the ovalbumin-inhaled mice model, [28], and TXM-CB3 in this system was effective at 10–50mg/kg [38]. TXM-CB3 also inhibited, at 1–10 mg/kg, inflammatory pathways in the brain of Zucker rats [39].

### A single dose of AD4 protects cognitive function in mice post mTBI

A single dose of AD4 (100mg/kg) administered 60 min post injury protected cognitive behavior of mice in two behavioral tests, the Y-maze and the NOR tests. Cognitive behavior was tested 7 and 30 days post injury. This protective effect is consistent with in vivo data demonstrating the ability of AD4 to restore cellular GSH [23], [24], prevent nuclear translocation of NF-KB in vivo [28], and inhibit the pro-inflammatory MAPK pathways [23], [36]. A smaller dose of AD4 (50 mg/kg) however, was ineffective at 7 days post injury, as shown in the Y-maze and in the NOR assay, and was ineffective in the recognition of novel object test 30 days post injury. Interestingly, in the thirty days post injury, cognitive performance was restored by 50 mg/kg AD4 as demonstrated in the Y-maze test.

Previously, treatment of unilateral controlled cortical impact (CCI) injury in rat model by continuous administration of AD4 showed tissue sparing, and protection from oxidative stress. In these studies, an AD4-loaded pump (18.5 mg/kg/hr) was used in addition to a single 150mg/kg bolus i.p injection, given 30 min post-injury [30]. Large continuous dosage (300mg/kg) of AD4 also maintained mitochondrial GSH at near normal levels. Treatment starting at 15min post-injury showed a significant protection maintaining acute mitochondrial bioenergetics and normalized GSH levels [30].

High doses of AD4 also reversed trauma-induced damage in the spinal cord. Mitochondrial bioenergetics were improved after spinal cord injury in rats receiving large doses of 75, 150, 300 or 600mg/kg, at 15 min and 6 h post-injury, with maximum effects at 300mg/kg [31].

More recently neuronal degeneration induced by focal penetrating TBI in male Sprague–Dawley rats was decreased by AD4 [32]. In these studies, rats were injected i.p with 300 mg/kg AD4 two-min post trauma, with additional bolus of 300 mg/kg i.p injected 4 hours later. The rats also showed an increase in the expression of Mn-superoxide dismutase [32]. The higher doses of AD4 required for the injury models noted above, compared to that needed for weight drop studies here, might be due to a greater severity of the initial injury after CCI or cord trauma.

### A single dose of TXM-CB3 or TXM-CB13 (DY-70) protects cognitive function post mTBI injury in mice

A potential treatment of mTBI, aimed at minimizing secondary brain injuries was further explored using Trx1 mimetic peptides (TXM-peptides). Trx proteins catalyze thiol-disulfide oxidoreductions by using redox-active cysteine residues within a -CxxC-motif. The association of reduced Trx1 into a complex with ASK1 blocks the enzymatic-activity of ASK1 in turn, inhibiting the apoptotic ASK-MEK-MAPK inflammatory pathway [48]. Therefore, the effects of TXM-peptides on the activation of the MAPK pathway were explored.

We showed that a single dose of TXM-CB3 (50 mg/kg) or TXM-CB13 (DY-70; 50 mg/kg) given 60 min post injury improved cognitive performance 7 days post-injury, as opposed to no protection offered by AD4 at the 50mg/kg dose. These results are consistent with the higher potency of TXM-peptides compared to AD4 in several cellular systems [36], [35], [38], [37]. The improvement in cognitive behavior by TXM-CB3 and TXM-CB13 (DY-70) was demonstrated in the Y-maze as well as in the NOR test 30 days post injury (**Fig 5**). TNF-α is one of the cytokines that has a major influence on the recovery post TBI. Previously we have shown, using our weight drop closed-head injury model, that TNF-α level increases around 12 h post injury [53]. Other TBI models also show that reducing post injury TNF-α levels is critical for recovery [54], [55], [56], [57]. Perhaps, lowering TNF-α levels by TXM-CB3, as shown in Zucker rat brain [39], contributes to the recovery of cognitive impairment post mTBI.

The cleavage of caspase3 is considered to have a major influence on apoptosis following traumatic brain injury [58]. Previously we showed a decrease in cleaved caspase3 after treatment with TXM-CB3, using a dose of 10mg/kg/day for 28 days in the Zucker rats [39]. Our results suggest that improved cognition behavior found using TXM-CB3 could result from inhibiting caspase3 cleavage. Other activities of TXM-peptides such as denitrosylation and oxidoreduction could also contribute to restoration of cognitive function [37], [40], [59].

TXM-CB13 (DY-70) inhibits the MAPK-apoptotic pathway induced by AuF as monitored by JNK, and p38^MAPK^ phosphorylation in human neuronal SH-5Y5Y cells. These results are similar to TXM-CB3 inhibition of MAPK activity [39] and further support the view that TXM-peptides could protect neuronal cells from mTBI by inhibiting neuronal inflammatory pathways.

In the Y-Maze test 30 days post injury, TXM-CB3 and AD4 improved cognitive performance, while TXM-CB13 (DY-70) was ineffective. In the NOR test only TXM-CB3 demonstrated a significant cognitive improvement. Hence, TXM-CB3 appeared to be the most effective in protecting visual and spatial memory both in the short and long-term period after injury. The decline in memory ability post mTBI observed in mice and the recovery induced by TXM-peptides is consistent with the need to lower inflammation at an early stage e.g. 60 min post trauma, in order to prevent secondary damage following injury [41], [53]. In view of the redox and anti-inflammatory activities of the different thiol reagents: TXM-CB3, TXM-CB13 (DY-70) > AD4 > NAC, we suggest that TXM-peptides could become effective in treating cognitive post mTBI impairment.

Further studies are required to establish and examine the potential use of a single application of TXM-peptide for preventing damage after brain trauma, for example, in chronic traumatic encephalopathy observed in American football players, which result from multiple concussions and other types of blows to the head. More work is also needed to understand the differences in drug efficacy observed between the Y-maze and the NOR test. Furthermore, it is essential to determine whether restoring short and long-term mTBI damage in cognitive function by TXM-peptides results from a structural advantage of the tri-peptide *vs*. the tetra peptide; or does it depend only on the redox potency of two Cys residues or on the amino acids composing the TXM motif. Finally, we aim to reveal the mechanism by which the anti-inflammatory and/or the denitrosylating activities of these potential drugs contribute to restoring post mTBI cognitive function.
